# Supplementary material for: Narrow bandwidth, low-emittance positron beams from a laser-wakefield accelerator
Source: Sci Rep. 2024 Mar 12;14:6001. doi: 10.1038/s41598-024-56281-1 (PMC10933426; doi:10.1038/s41598-024-56281-1)
Supplement: Supplementary file 1 — Supplementary Information. [file 41598_2024_56281_MOESM1_ESM.pdf]

Supplemental Information for "Narrow bandwidth, low-emittance positron beams from a laser-wakefield accelerator".

February 12, 2024

1 Experimental setup

The setup for the experiment described in this paper is shown in figure S1.

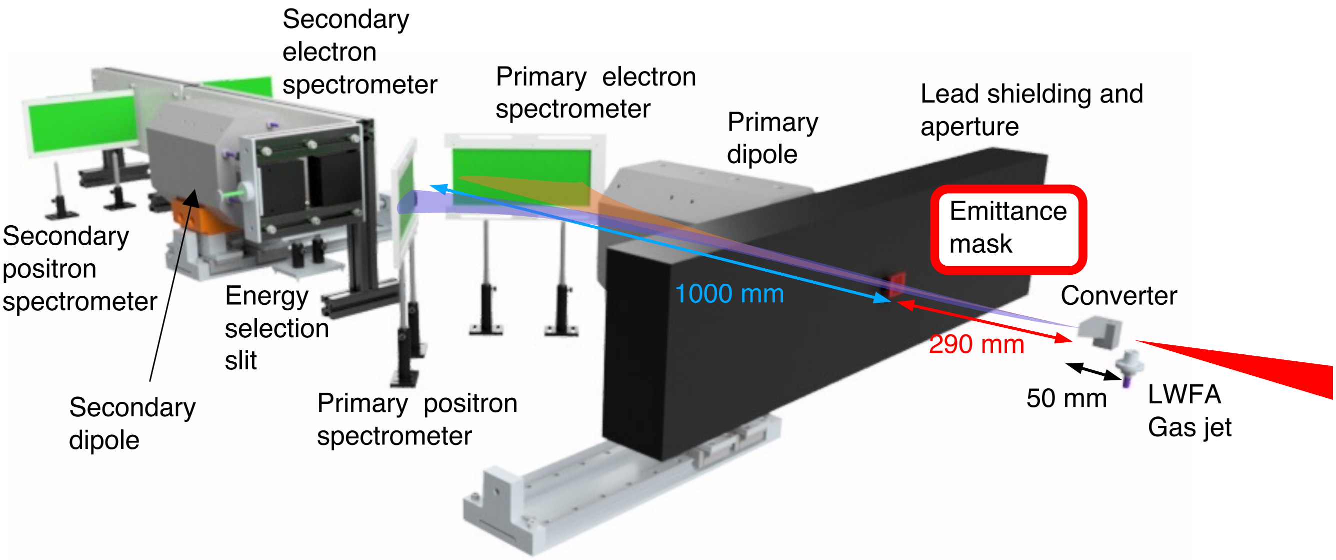

Figure S 1 – Schematic of the experimental setup. The laser is focused into the gas jet to drive the Laser-Wakefield Accelerator which generates the primary electron beam. This electron beam generates electron-positron pairs in the converter which then propagate through an aperture in the lead wall. The primary dipole disperses the electron and positron beams (positrons shown in blue) onto the spectrometer screens. The emittance mask can be placed into the beam to measure the electron and positron beam spatial properties. A secondary electron spectrometer screen is used to improve the accuracy of the electron spectrum measurement. The secondary dipole and positron spectrometer screen is used to be perform energy selection.

2 Two-screen electron spectrometer

Two screens were placed in the electron beam after the magnetic dipole, with some drift space in-between. The signal seen on each screen is due to the energy and angular spectra of the electron beams generated in the plasma. It is assumed that all electrons were generated at a fixed point at the exit of the LWFA. Electrons with kinetic energy in the range of $100 \geq \gamma m_e c^2 \geq 2500\,\mathrm{MeV}$ and propagation angles relative to the design axis of $-15 \geq \theta_y \geq 15\,\mathrm{mrad}$ were numerically tracked through the magnetic field of dipole, and the location of their intersections with the LANEX screens was recorded. This was used to produce a look-up table which gave the particle energy as a function of its position on each screen and its initial propagation angle θ_y. The electron beam spectrum was determined by finding the coefficients of a third-order polynomial

function $\theta_y(\gamma)$ that minimised the mean squared difference between the retrieved angularly integrated electron spectra from each screen.

Charge calibration of the electron spectrometers were performed by measuring electron spectra on an image plate placed in front of the second LANEX screen and comparing to the images recorded on the CCD over the same shots. The image plate used was BAS-TR2040, with a sensitivity of 1 PSL per 350 electrons. The first electron screen was then calibrated by matching the angularly integrated spectra recorded on each screen. A retractable LANEX detector was placed close to the exit of the first dipole magnet so that it could observe both the positron and electron beams with a single CCD. This was used to measured the relative yield of positrons and electrons at the same time as observing the signals on the primary electron and positron screens. The relative yield was then used to cross-calibrate the charge sensitivity of the primary positron screen.

3 Lepton source size, divergence and emittance retrieval

The beam profile after the beam aperture was modelled as a an azimuthally symmetric clipped Gaussian distribution, such that only particles with $x_{i,\text{ap}}^2 + y_{i,\text{ap}}^2 \leq R_{\text{ap}}^2$ were transmitted, where $x_{i,\text{ap}}$ and $y_{i,\text{ap}}$ are the transverse spatial coordinates of the ith particle at the aperture plane z_{ap}, with aperture radius R_{ap}. This profile was dispersed according to the individual particle energies onto the spectrometer screen. The particle distribution $S_y'(x)$ was measured at the detector plane z_{det} where x and y are transverse coordinates perpendicular and parallel to the dispersion plane of the spectrometer respectively. Due to the combination of energy spread and divergence, the profile $S_y'(x)$ is due to particles over a range of different energies where their initial propagation angle $\theta_{i,y}$ and energy E_i result in the particle intersecting the detector plane at the position y. With the assumption that the spectrum $N(E)$ is slowly varying, then each slice measurement $S_y'(x)$ represents the integral of the beam profile over y, i.e.,

$$
\begin{aligned}
S_y'(x) &= 2A_{y,0} \int_0^{\sqrt{R^2-x^2}} \exp\left[-\frac{x^2+y^2}{2\sigma_x^2}\right] \mathrm{d}y \\
S_y'(x) &= \sqrt{2\pi} A_{y,0} \sigma_x \,\mathrm{erf}\left(\sqrt{\frac{R^2-x^2}{2\sigma_x^2}}\right) \exp\left[-\frac{x^2}{2\sigma_x^2}\right]
\end{aligned} \tag{1}
$$

where $A_{y,0}$ is the amplitude of the particle distribution, $R = R_{\text{ap}} z_{\text{det}}/z_{\text{ap}}$ is the projected size of the aperture at the detector plane and $x \leq R$. The functional form of equation 1 was used to fit the amplitude of the modulated signal $S_y(x)$ when retrieving the apertured beam properties as described below.

Several steps were followed to extract the particle emittance from the spectrometer signals. Firstly, a variable threshold filter was used to remove hard-hits caused by stray photons hitting the CCD directly. Secondly, the defocusing of the effect of the magnetic dipole fringe fields was removed by re-scaling the measured signal in non-dispersion direction such that the spatial frequency of the grid pattern was made constant for all energies. Then vertical slices were taken through the resultant image, averaging over 4 mm in the dispersion direction to produce the signal modulation $S_y(x)$ as a function of x at a given $y-$position. The scattered particles from the grid formed a smooth background on the detector which was removed by fitting a Gaussian to the values at the minima of the observed modulations. The envelope of the signal was similarly found by fitting the beam profile function (equation 1) to the signal maxima. The RMS width of the fitted envelope was then divided by the source-to-screen distance to obtain the beam divergence σ_θ.

An ideal zero source size beam would produce a sharp step-function within the bounds of the scattering signal and the beam envelope, with the spatial period of the magnified grid size. Blurring of this pattern was observed due to contributions of the finite spatial resolution of the diagnostic (215 μm) and the source size σ_x of the beam, which was found by iterative minimisation of the mean squared error between the measured signal and the calculated signal for a given source size. The geometric emittance was calculated as the product of the measured divergence and source size, i.e. $\epsilon = \sigma_x \sigma_\theta$. An example measured signal and retrieved modulation signal is shown in figure S2.

In order to benchmark the retrieval process, synthetic data was created by numerically propagating results from a FLUKA simulation and removing particles that would hit the solid bars of the emittance

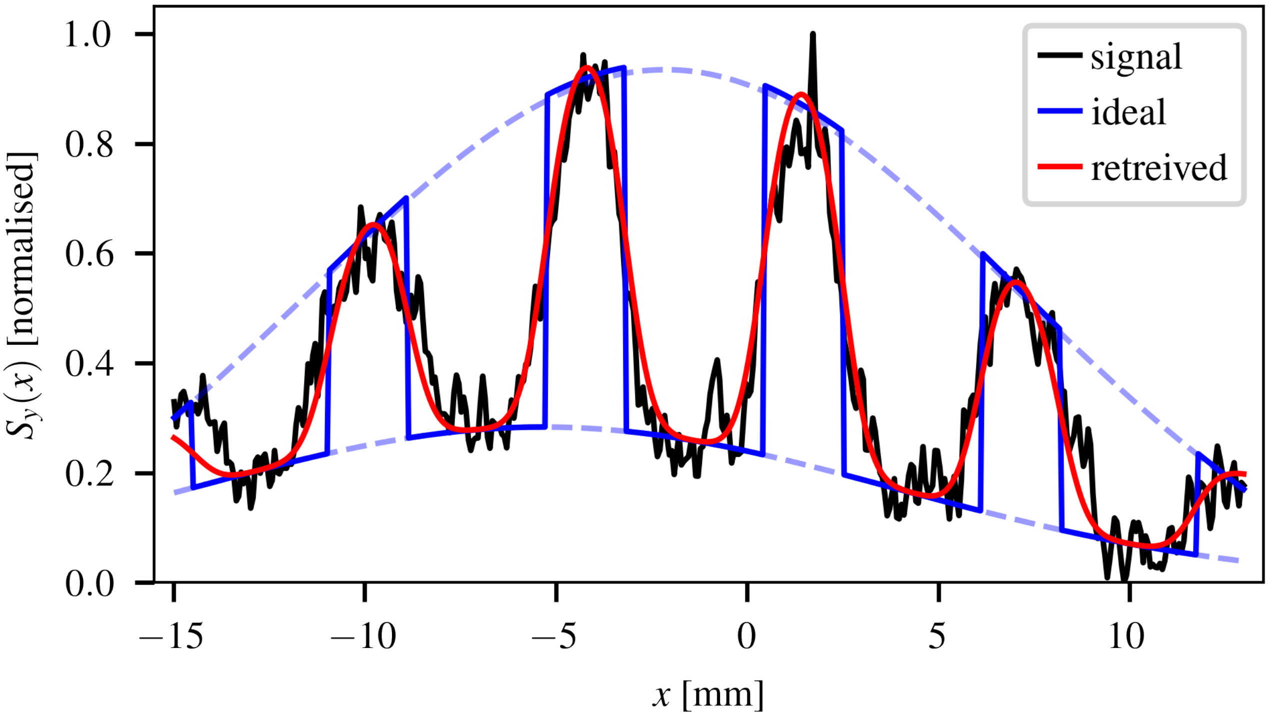

Figure S 2 – Example signal modulation fitting for beam parameters retrieval. The signal (black line) is taken for a central positron energy of 420 MeV with a converter thickness of 8 mm. The retrieved signal (red line) corresponds to a source size of 127 μm. An ideal beam (zero source size) would produce a rectangular profile pattern (blue line) between the scattered signal and the beam amplitude (blue dashed lines).

measurement grid. The dispersion of the magnet was added by shifting the particles transversely according to their energy using the same dispersion function as for the experimental spectrometer. To create the modulated signal $S_x(y)$ for a given energy band, the particles are selected according to their position on the spectrometer. Due to the significant beam divergence, there is some trajectory crossing such that some particles of different energies are selected, and some of the correct energy are omitted, as illustrated in figure S3.

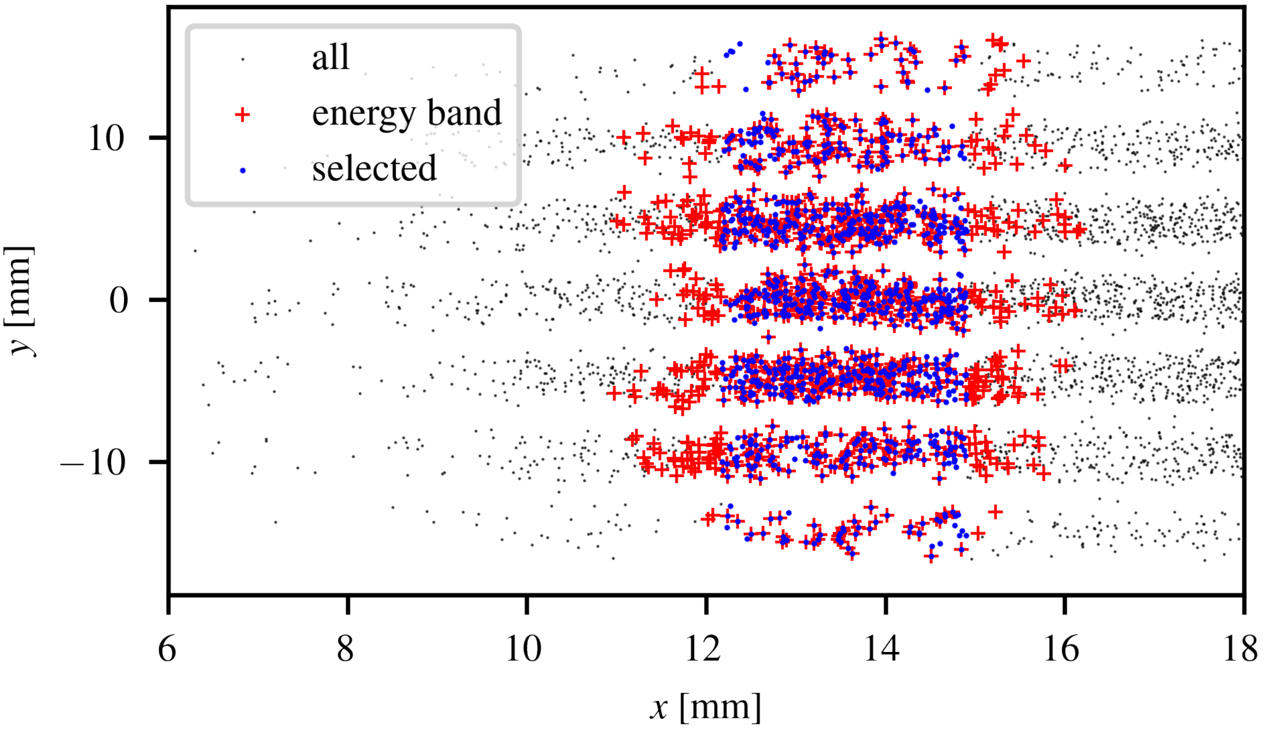

Figure S 3 – Example synthetic positron spectrometer particle distribution. Particles that pass through the grid were propagated ballistically onto the detector plane, and then dispersed according to their energy. Taking a slice along the y-axis mixes some particle energies together due to the effect of the beam divergence. Particles label 'energy band' have energies within 450-550 MeV. Selected particles have x positions corresponding to the expected position for zero-divergence particles within the same energy range. The signal $S_x(y)$ is generated by making a histogram of selected particle y coordinates.

The synthetic signals were analysed with the same procedure as for the experimental data and compared to the values directly calculated from the particle distributions, as shown in figure S4. The retrieved beam properties closely agree with the directly computed values for the apertured beam, verifying the analysis procedure.

4 FLUKA simulations

Simulations of the bremsstrahlung induced pair-production process were performed using the particle physics Monte-Carlo code FLUKA with the EM-cascade defaults. Electrons were initialised with zero source size and transverse momentum from an energy distribution chosen to match the experimentally measured average LWFA spectrum. A lead converter of variable thickness L was placed in the path of the electrons, and the momenta and position of all electrons, positrons and photons were recorded as they exited the rear surface of the converter. The simulations were performed for 10^6 primary electrons for each converter thickness.

In order to simulate the effects of the finite divergence of the electron beam, each particle was assigned random angular shifts $\Delta x_i'$ and $\Delta y_i'$) from the probability density function $f(x') = f_0[(x'/\theta_w)^2 + 1)]^{-2}$,

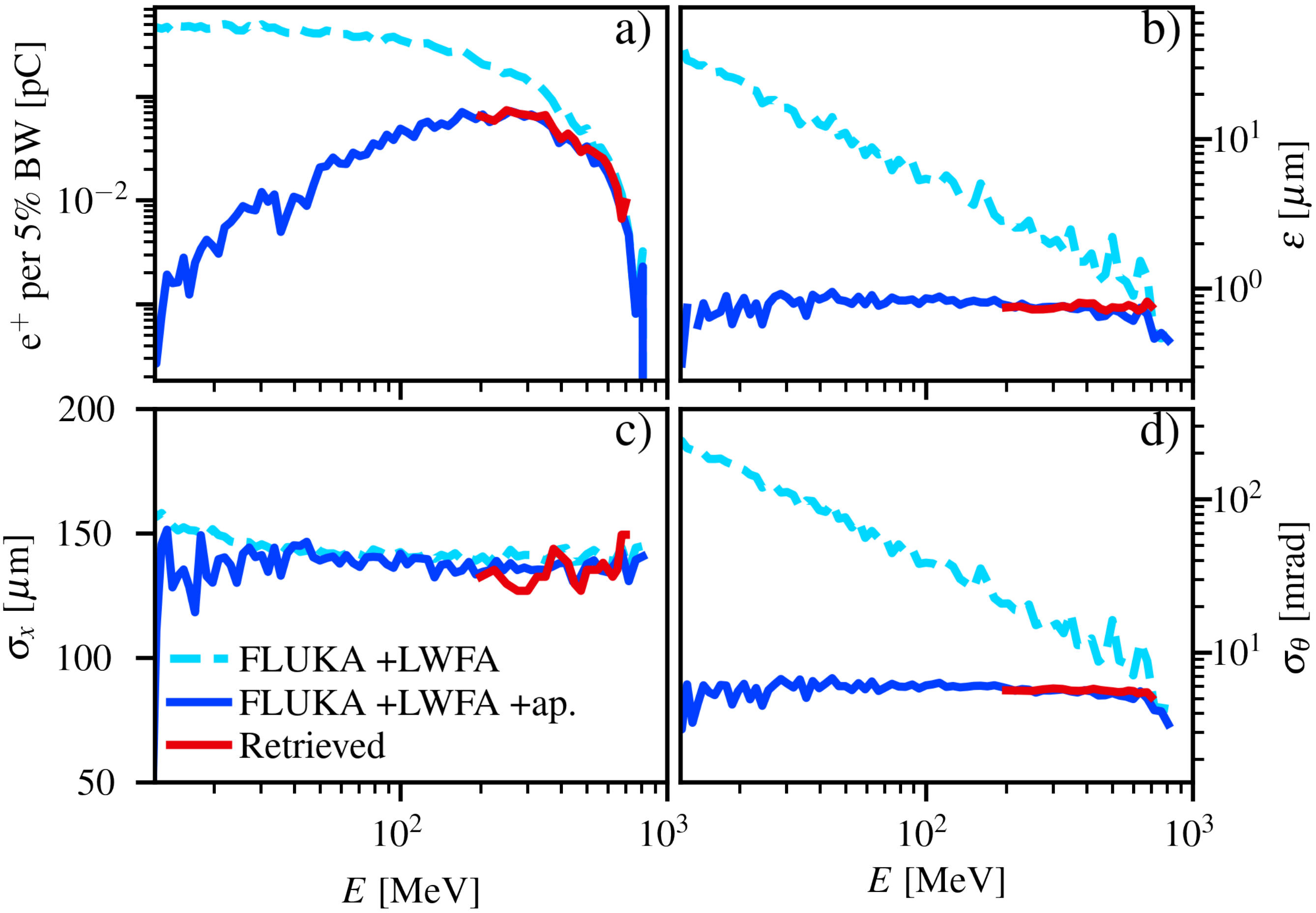

Figure S 4 – Comparison of retrieved positron beam a) spectrum, b) emittance c) source size and d) divergence with values computed directly from the particle distributions. Properties are plotted for: the synthetic diagnostic data (red line); the FLUKA particle distribution including the effects of finite primary electron beam divergence and source size (cyan dashed line); the FLUKA particle distribution also clipping the beam with a 12.6 mrad aperture (blue line). The FLUKA simulation used a 1 mm thick lead converter.

which was seen to approximate the experimentally transverse profile of the primary electron beam with $\theta_w = 2.9 \pm 0.3$ mrad (f_0 is the normalisation constant). The particle transverse momenta and positions were then altered according to these shifts and using the experimental drift length between the LWFA exit and the converter rear face of 50 mm. The transverse particle positions were also modified according to the expected LWFA electron source size of 1 μm, although this contribution was negligible. Each particle was shifted 10 times from the value taken from the FLUKA simulation, with the shifted particle properties recorded each iteration to produce a final list with 10 times the number of particles as were produced by the FLUKA simulations. Particle distribution properties were then calculated at the longitudinal plane for which the correlation term $\langle xx' \rangle$ was minimised (typically fractionally inside the rear surface of the converter).

5 FBPIC simulations

FBPIC simulations were performed to explore the acceleration of the generated positron beam in a laser wakefield accelerator as described in the main article. In addition to the results presented there, a simulation was also ran with an energy selected bunch. Only the positrons with energies of 500±50 MeV were initialised in the simulation, as shown in figure S5a and S5c. Of the charge initialised in the simulation, 46% was trapped and accelerated to higher energies ($E > 0.8$ GeV) with the average energy increasing to 1.2 GeV. The remainder was comprised of particles which were initialised too far from the central axis of the laser to become trapped. A strong chirp developed in the longitudinal phase space (figure S5d) as a result of the longitudinal variation of the plasma wakefield. Initially, the normalised emittance rapidly increases due to mismatch between the beam and the plasma. From 10 mm onwards, the normalised emittance was stable at 60 µm.

FLUKA simulations for different target thicknesses

Simulations were performed to estimate the positron beam properties produced in the interaction of the primary electron beam with the converter target. The effects of the beamline were then calculated and compared to the experimental results, as shown in the main paper for the 1 mm thick converter. The results for the 1 mm, 2 mm, 4 mm and 8 mm are shown here in figure S6.

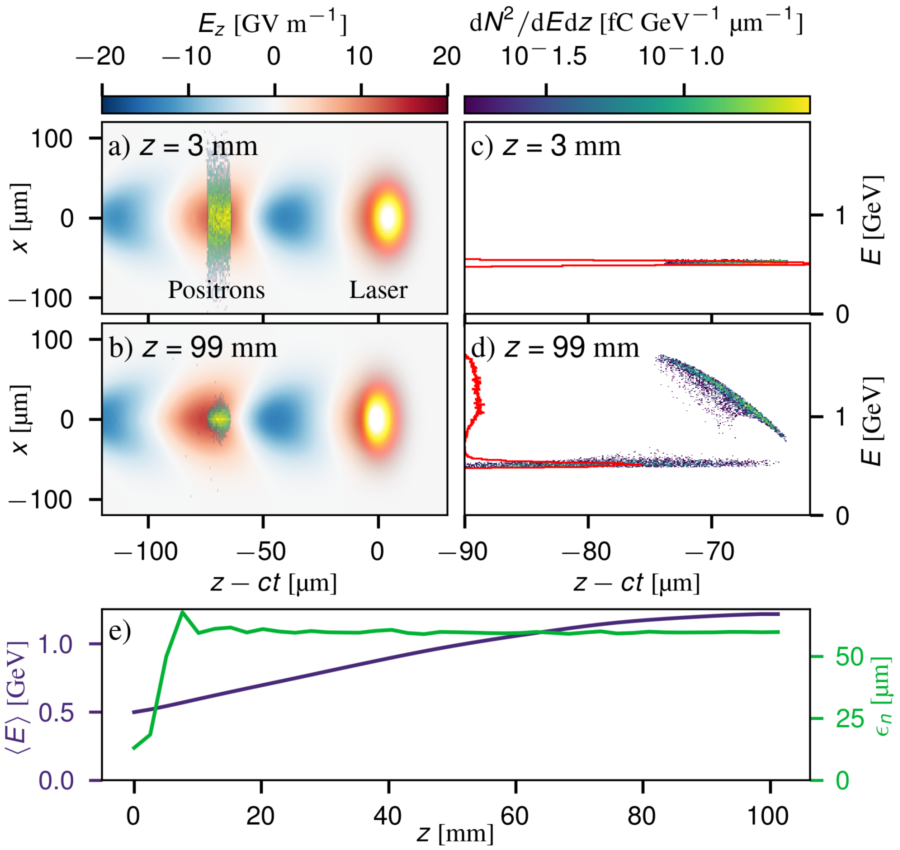

Figure S 5 – **Simulated post-acceleration of the experimentally energy-selected positron bunch.** **a&b** show the longitudinal electric fields of the plasma wakefield generated by the laser pulse (yellow orb) and the trailing positron bunch at the beginning of the plasma and after 96 mm of propagation. Panels **c&d** show the positron longitudinal phase space before and after acceleration, with the energy spectra indicated by the red lines. **e** shows the average energy and normalised emittance of the trapped bunch, defined as being comprised of particles which remain within ±50 μm of the central axis.

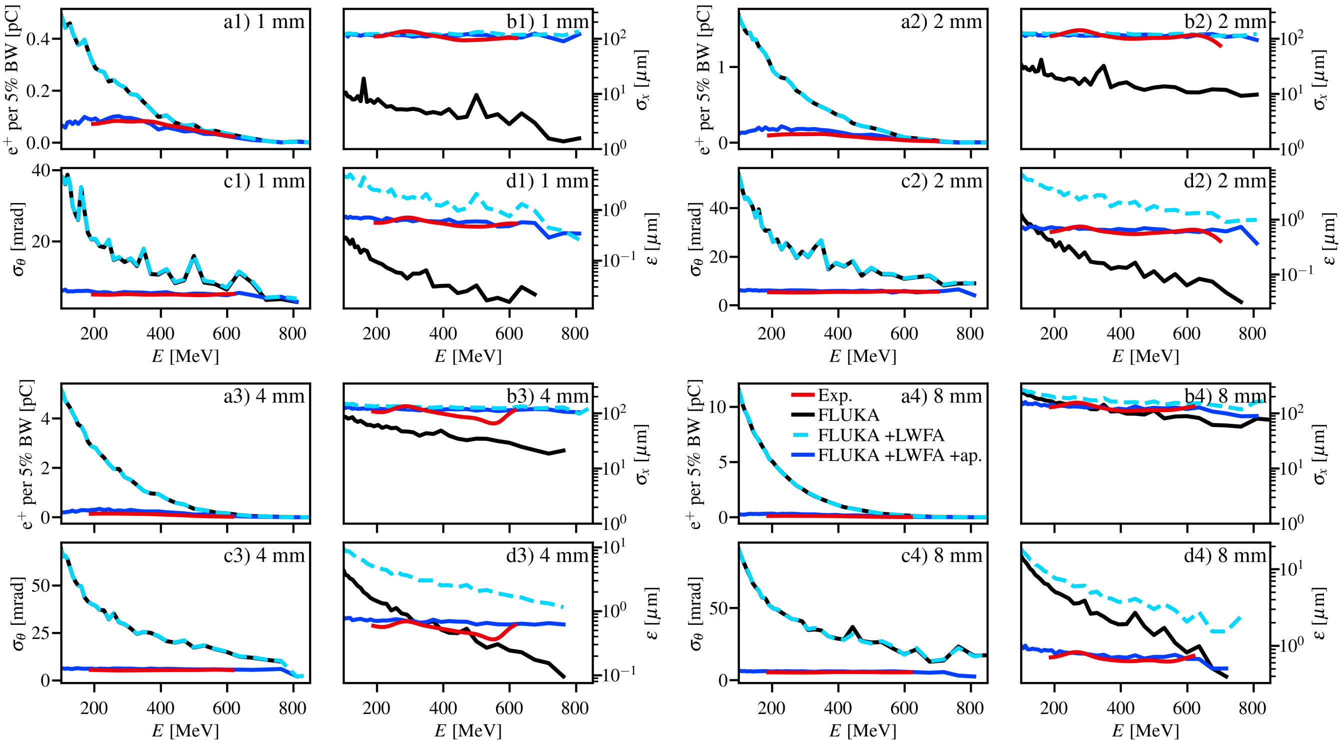

Figure S 6 – **Comparison with numerical simulations.** Positron beam a) spectrum (charge per 5% bandwidth), b) source size, c) divergence and d) geometric emittance plotted for **1**) 1 mm, **2**) 2 mm, **3**) 4 mm and **3**) 8 mm converters. The experimental data (red) is plotted alongside FLUKA simulations for: zero drift distance for the primary electron beam (black); including the drift distance and the primary electron beam divergence and source size (cyan dashed); and including the 12.6 mrad shielding aperture (blue).
